# Implementing diffusion-weighted MRI for body imaging in prospective multicentre trials: current considerations and future perspectives

**DOI:** 10.1007/s00330-017-4972-z

**Published:** 2017-09-27

**Authors:** N. M. deSouza, J. M. Winfield, J. C. Waterton, A. Weller, M.-V. Papoutsaki, S. J. Doran, D. J. Collins, L. Fournier, D. Sullivan, T. Chenevert, A. Jackson, M. Boss, S. Trattnig, Y. Liu

**Affiliations:** 1 CRUK Cancer Imaging Centre, Institute of Cancer Research and Royal Marsden NHS Foundation Trust, Downs Road, Surrey, SM2 5PT UK; 2 Manchester Academic Health Sciences Institute, University of Manchester, Manchester, UK; 3 Assistance Publique-Hôpitaux de Paris, Hôpital Européen Georges Pompidou, Radiology Department, Université Paris Descartes Sorbonne Paris Cité, Paris, France; 4 Duke Comprehensive Cancer Institute, Durham, NC USA; 5 Department of Radiology, University of Michigan Health System, Ann Arbor, MI USA; 6 Applied Physics Division, National Institute of Standards and Technology (NIST), Boulder, CO USA; 7 Department of Biomedical Imaging and Image guided Therapy, Medical University of Vienna, 1090 Vienna, Austria; 8 European Organisation for Research and Treatment of Cancer, Headquarters, Brussels, Belgium

**Keywords:** Diffusion-weighted MRI, Multicentre trials, Quality assurance, Quantitation, Standardization

## Abstract

**Abstract:**

For body imaging, diffusion-weighted MRI may be used for tumour detection, staging, prognostic information, assessing response and follow-up. Disease detection and staging involve qualitative, subjective assessment of images, whereas for prognosis, progression or response, quantitative evaluation of the apparent diffusion coefficient (ADC) is required. Validation and qualification of ADC in multicentre trials involves examination of i) technical performance to determine biomarker bias and reproducibility and ii) biological performance to interrogate a specific aspect of biology or to forecast outcome. Unfortunately, the variety of acquisition and analysis methodologies employed at different centres make ADC values non-comparable between them. This invalidates implementation in multicentre trials and limits utility of ADC as a biomarker. This article reviews the factors contributing to ADC variability in terms of data acquisition and analysis. Hardware and software considerations are discussed when implementing standardised protocols across multi-vendor platforms together with methods for quality assurance and quality control. Processes of data collection, archiving, curation, analysis, central reading and handling incidental findings are considered in the conduct of multicentre trials. Data protection and good clinical practice are essential prerequisites. Developing international consensus of procedures is critical to successful validation if ADC is to become a useful biomarker in oncology.

***Key Points*:**

• *Standardised acquisition*/*analysis allows quantification of imaging biomarkers in multicentre trials*.

• *Establishing* “*precision*” *of the measurement in the multicentre context is essential*.

• *A repository with traceable data of known provenance promotes further research*.

## Essentials

1. When utilizing the Apparent Diffusion Coefficient (ADC) as an imaging biomarker in multicentre trials, processes that standardise data acquisition and analysis within a framework of Quality Assurance and Quality Control are mandatory.
2. Test-object and healthy volunteer studies should be used to develop an imaging protocol for multi-vendor, multi field-strength use and establish the precision of the ADC measurement within a multicentre trial context.
3. A streamlined workflow for data curation, archiving and analysis in a central repository ensures traceable data within the trial as well as its preservation for further research.

## Patient Impact

1. A standardised ADC measurement would enable incorporation of an imaging biomarker of response as an early end-point in multicentre trials of cancer therapies.
2. A standardised ADC measurement in longitudinal studies could be utilized as a prognostic biomarker in oncology and for stratifying patients for therapeutic interventions.

## Introduction

Diffusion-weighted magnetic resonance imaging (DW-MRI) provides unique soft tissue contrast and is now used in tumour detection, staging and for monitoring response to treatment in a variety of tumour types [1–8]. It may be utilized qualitatively (binary, normal vs. abnormal), semi-quantitatively (scoring system, e.g., grade I-V) or quantitatively (continuum, derived numerical values). Qualitative assessments are quick and easy for the expert radiologist but are variable in interpretation. Objective semi-quantitative (scoring systems) or quantitative (numerical) assessments are more robust; the latter deliver information beyond visual perception.

The apparent diffusion coefficient (ADC) derived from DW-MRI describes the diffusion of a water molecule proton (typically over 10-40 μm during 10-100 msec) and reflects tissue microstructure and its remodelling. This is interesting for drug developers as it sits in the “pharmacologic audit trail” [9] downstream of a target and its pathway (thereby uniting many therapy classes), but upstream of macroscopic disease modification (thus making it suitable for early readouts). Such quantitative measurements potentially offer earlier indicators of response than conventional size criteria, with ethical and economic benefits for sponsors and pharmaceutical companies as well as for patients and society in general. The implementation of DW-MRI, however, is variable across scanner platforms [10], tissue-type being studied and methods of interpretation and analysis. Consensus on image acquisition and analysis methods must be reached before embarking on a clinical trial and measures put in place to standardise the process across centres. Furthermore, the utility of quantitative ADC metrics as response biomarkers depends on the variability of the measurement, which must be established and minimized. This article reviews current knowledge of factors that require consideration (equipment, technical development, quality control, infrastructure, expertise and governance issues) when acquiring and analysing DW-MRI data prior to adopting ADC as a biomarker in multicentre trials.

## Data Acquisition

### Hardware and software considerations

Over the last decade significant hardware improvements have enhanced data acquisition. Signal-to-noise ratio [SNR] improvements have resulted from higher field strength (3T), improved magnetic field gradient performance (increased maximum gradient amplitudes and ramp rates), improved digital radiofrequency (RF) chains and receiver technology with multiple receiver arrays. Advanced digital compensation schemes further mitigate gradient-induced eddy currents reducing image distortion and blur. Although DW-MRI at 3T initially struggled to match the quality of large field-of-view (FOV) 1.5T DW- MRI images because of inhomogeneity of the static magnetic field (B0), recent advances in automated correction (shimming) and improvements in static field homogeneity have made modern 3T platforms viable options for body imaging. In normal volunteers, ADC values of upper abdominal organs are comparable across field strengths; however, the coefficient of variation, (CoV) of the liver was 1.5 - 2.0 times greater at 3.0T compared to 1.5-T [11], emphasising that suitability for inclusion in a multicentre trial requires assessment of individual scanner performance.

### Optimising a DW-MRI protocol

Protocol optimisation is often scanner-specific as available measurement and artefact [12] reduction techniques vary between manufacturers, models and software versions. Geometric distortion associated with the static magnetic field can be reduced by using methods to correct field inhomogeneity (advanced shimming) and by increasing the readout bandwidth [13–15]. Distortions arising from eddy-currents can be diminished by reducing the diffusion-weighting (maximum b-value) and other sequence parameters (echo-train length, matrix) or by employing gradient schemes such as the twice-refocused spin echo [16] that compensate for eddy-currents, as well as by using post-acquisition image registration routines [17]. Ghosting artefacts (displaced re-duplications of the image) can be reduced by adjusting the receiver bandwidth and echo time.

**Fig. 1:**
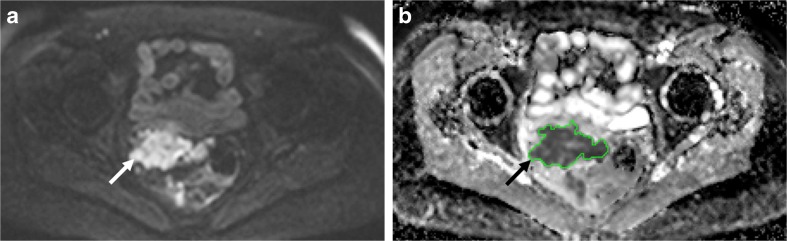
Diffusion-weighted-MRI in relapsed peritoneal cancer: Axial b-value=900 mm^2^/s (A) and image through the mid pelvis showing an irregular mass (arrow), with restricted diffusion contoured using a semi-automated region-growing tool. The tumour shows relatively limited signal decay with increasing b-value on the apparent diffusion coefficient map (B), and appears dark compared to normal tissues (arrow)

Depending on the disease, an optimal selection of b-values [18] is needed with considerably more b-values required if the signal decay with increasing b-value is to be fitted to non-mono-exponential functions [19]. To avoid confounds from perfusion, b-values of <100 s/mm^2^ should be avoided: maximum b-values of 800 to 1000 s/mm^2^ are usual in body applications (Fig. 1) [20] but their range may need optimisation for specific tumour types. Noise characteristics influence the maximum b-value used in practice. The number of signal averages may be increased at higher b-values to increase SNR [21]. Most DW-MR images are acquired in free-breathing, averaging the signal over physiological motion. Respiratory triggering, using bellows or a navigator, has not shown advantages over multiple averaged free-breathing in estimation of ADCs in abdominal organs [22, 23]. Cardiac triggering has been explored in the upper abdomen [24]. Anti-peristaltic agents reduce image blur arising from peristaltic motion in abdominal and pelvic DW-MRI and multishot techniques may offer some advantages over single-shot techniques in reducing distortion from air within bowel [25].

Parallel imaging reduces geometric distortion, but reduces SNR. The extent of the imaging volume along the scanner bore (z-axis) should be limited to around 25 cm (depending on scanner capability) to mitigate bias in ADC estimates due to spatial non-linearities in diffusion-encoding gradients [26]. For larger volumes, multiple imaging stations can be acquired at the isocentre of the magnet sequentially [27]. Acquisition of multiple stations requires software tools to normalize station-to-station signal variation and the ability to compose the images into a single series for a given diffusion-weighting (b-value). At 1.5T, spectral fat-suppression techniques are often used for abdominal, pelvic or small FOV applications, while inversion recovery is used for whole-body DW-MRI and in regions of poor static magnetic field homogeneity. Fat suppression at 3T is more challenging, and the preferred method may vary between scanners; combinations of suppression techniques may be required [28]. Some consortia such as the Quantitative Biomarkers Imaging Alliance (QIBA) and the European initiative Quantitative Imaging in Cancer-Connecting Cellular Processes to Therapy (QuIC-ConCePT) have been working on standardisation and optimisation of DW-MRI acquisition protocols, and technically validated protocols, e.g., in liver and lung are available to the public [29, 30].

In multi-centre trials, compromises may be required in acquisition parameters in order to achieve an acceptable degree of standardization whilst maintaining good image quality on all scanners [12]. A current list of multicentre trials reporting DW-MRI as a readout in body imaging applications is listed in Table 1.

**Table 1:** Published multicentre DW-MRI clinical studies

Cancer	N	Treatment	Field strength/Parameter	Gold- standard	Performance	Repeatability/Rep reducibility	QA/QC
Technical validation							
Locally advanced breast cancer [31]	4 centres; 54 patients	NA	1.5T b-values 0, 100, 600, 800 s/mm^2^	NA	Evaluation of Gradient nonlinearity correction (GNC)	Mean ΔADC =9.42% without GNC vs 9.41% with GNC (differences up to ±4% in individual pts)	Phantoms: with GNC overall mean error for all sites = 0.6% (without GNC = 9.9%)
Characterization							
Malignant musculoskeletal tumours (4 lymphoma, 11 metastases, 26 sarcomas) [32]	4 centres; 51 patients	NA	1.5T b-values 0, 1000 s/mm^2^ Whole tumour ADC	Pathology	Muscle lymphoma showed statistically significant lower ADC values	None	None
Staging							
Hodgkin’s or Non- Hodgkin’s Lymphoma [33]	3 centres; 108 patients	NA	1.5T Qualitative 2 readers	Bone marrow biopsy, FDG-PET, and follow-up	(Ann Arbor Classification)	=0.51	None
Cervical cancer [34]	2 centres; 68 patients	NA	3T ADC b-values 0, 150, 500 and 1000 s/mm^2^	Pathology	ADC not significantly different in metastatic nodes	2 Observers No reproducibility reported	Consensus for qualitative evaluation
Treatment response							
Solid tumours (phase I) [35]	2 centres; 13 patients	Combretast atin A4 phosphate and bevacizum ab	1.5T - ADC total (b-values 0, 50, 100, 250, 500, 750 s/mm^2^), - ADC high (b- values 100, 250, 500, 750 s/mm^2^) - ADC low (b-values 0, 50, 100 s/mm^2^)		Significant increase in median ADC total and ADC high 3 h after the second dose of CA4P	Repeatability (baseline examination) ADC total = 13.3% ADC high = 14.1% ADC low = 62.5%	Sucrose phantom measured at the two sites at 22°C
Locally advanced rectal cancer [36]	3 centres; 38 patients	Preoperativ e chemoradi ation	?T b-values 0, 1000 s/mm^2^	Pathologic al complete response (pCR) rate	ADC increased by 44.5% in pCR group, and decreased by 7.6% in non-pCR group (*P* = 0.026)	None	None
Locally Advanced Rectal Cancer [37]	3 centres; 120 patients	Chemoradi ation	1.5T - Qualitative (on b-value 1000 s/mm^2^) -- 3 readers	Pathology tumour regression grade (TRG)	Sens = 52-64% Spec = 89-97% ROC AUC = 0.78- 0.80	Inter-observer agreement =0.51-0.55	None
Locally advanced rectal cancer [38]	2 centres; 112 patients	Chemoradi ation	1.5T Qualitative (b-values 1000–1100 s/mm^2^) DWI post-treatment volume (manual) 2 readers	Pathology tumour regression grade (TRG)	Sens = 70% Spec = 98% ROC AUC = 0.92	Intraclass corre- lation coefficient (ICC) = 0.72-0.81	None
Locally advanced breast cancer [2]	3 centres; 39 patients	Chemother apy	3T, b-values 0-800 s/mm^2^		PRM ROC AUC at 8-11 days = 0.964	test-retest for repeatability (13	thermally controlled diffusion
	retrospective		Histogram analysis and voxel-based Parametric Response Map (PRM)		Whole tumour ADC ROC AUC at 35 days = 0.825	pts, 1 centre) ≤ ±0.1x10^-3^mm^2^/s.	phantom (1 centre)

NA: not applicable ; ADC: Apparent Diffusion Coefficient ; ROC: Receiver Operating Characteristic; AUC: Area Under the Curve

NB Current trials entered on clinicaltrials.gov are not included here

### Setting up Quality Assurance: Test Objects

According to metrology standards of quantitative imaging biomarkers (QIB) [39], measurement performance should be evaluated by assessing repeatability, reproducibility, linearity and metrics of bias. Test-object measurements yield practical estimates of the bias and the repeatability of each clinical MRI system and can be used to compare technical accuracy across the systems [40]. Precise measurement of ADC is important since the dynamic range of the biomarker is quite small, from approximately 0.5×10^-3^ mm^2^/s in densely packed cells to 3×10^-3^ mm^2^/s in fluid-filled cysts.

**Fig. 2:**
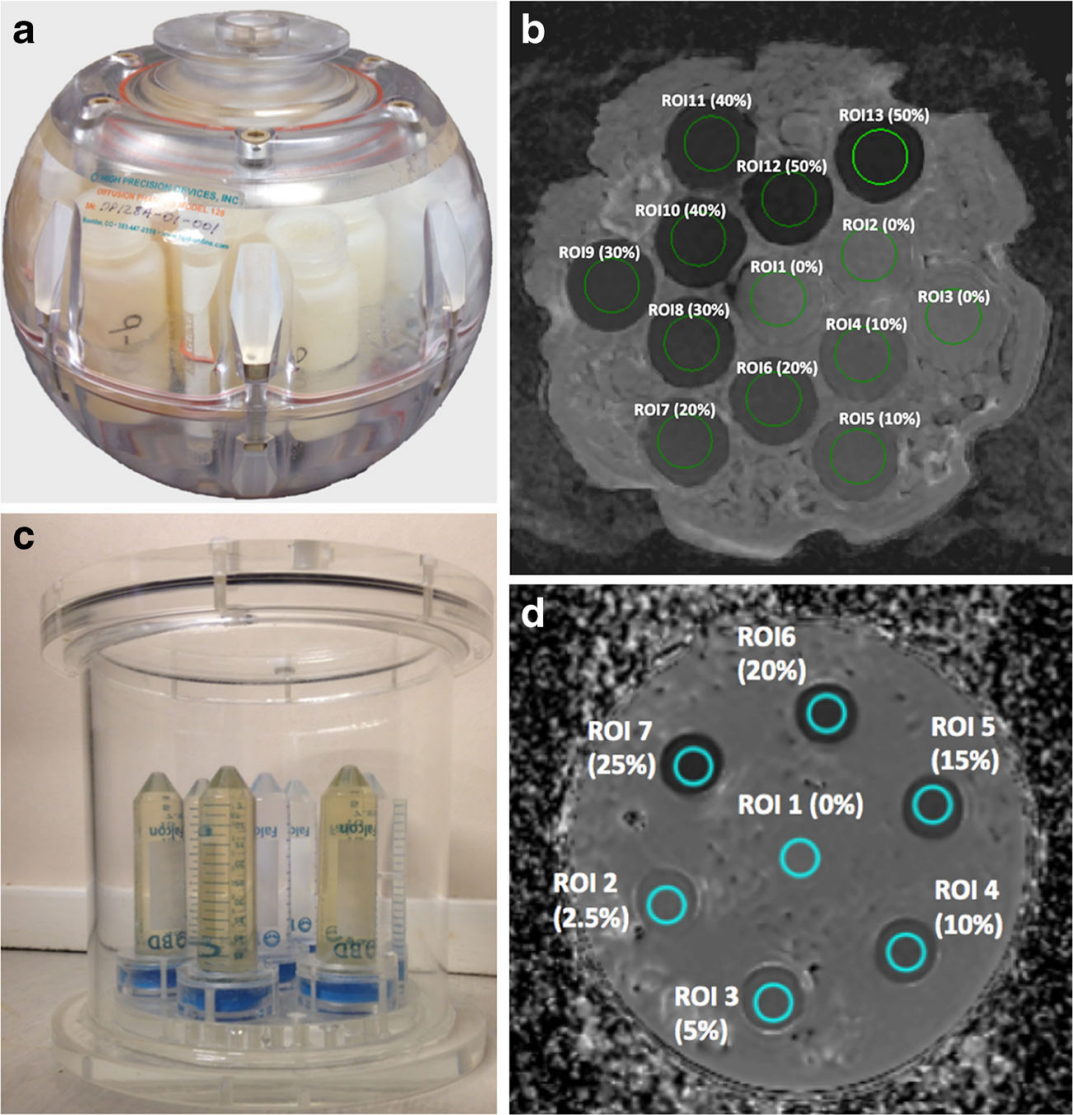
Test-objects for Quality Assurance in diffusion-weighted imaging: Spherical PVP phantom produced by QIBA and NIST (A) and corresponding axial ADC map (B); Cylindrical PVP phantom produced at The Institute of Cancer Research UK and used for EU multicentre trials within the QuicConCePT consortium (C) with the corresponding ADC map (D). The regions of interest in B and D denote the concentration (volume/volume) of PVP in water

Ice-water test-objects comprising multiple tubes with distilled water at 0°C and one of sucrose solution [30, 41] have been used but did not provide a sufficient range of ADC estimates. Following this, an ice-water test-object containing multiple sucrose samples doped with metals to reduce relaxation times to physiological values was presented [42] and utilized [12] for optimising a diffusion-weighted protocol in a multi-centre setting. Solutions of polyvinylpyrrolidone (PVP) in water embedded in an ice-water filled sphere [43] or cylindrical vessel [44], remain limited in their range of ADCs (Fig. 2). More specific test-objects have assessed ADC uniformity [12], ghosting and distortions [45, 46].

Test-objects at room temperature are more convenient to prepare than those with ice-water and have been used in single-centre studies [47, 48] but require correction from a temperature-controlled experiment [47] to account for temperature dependence of ADC. The performance of routine test-object evaluations in multi-centre trials involving DW-MRI, their frequency and pass-fail criteria, depends on the trial design and the nature of the imaging endpoint. Test-objects with the required range of ADCs need to be supplied and utilized at participating centres.

### Role of Healthy volunteer studies

Test-objects lack the necessary variation in tissue structure, geometry and motion experienced when imaging humans. Therefore, several trials have built in normal volunteer assessments during set-up.

Optimisation [49] and refinement of the DW-MRI measurement, e.g., multiband techniques [50], Zonal Oblique Multislice [51] and diffusion tensor imaging (DTI) can be assessed [52]. Protocols can also be tailored to underlying tissue structure e.g., lower b-values in pancreas [53] and tolerable versions for clinical use can be developed. Data analysis methods can also be optimised by seeking the best model fits of data from normal tissue [54] which can then be used as a comparator with pathological tissue to investigate structural differences [55].

Healthy volunteer studies are also useful for establishing physiological variation and reference values for disease e.g., in liver [56], bladder [57], bone marrow [58] and breast [59, 60].

Finally, normal volunteer studies are invaluable for studying technique repeatability: coefficient of variation of mean or median ADC estimates in breast 8%,[61] in liver 5.1% [62] and in skeleton 3.8% [63] have been reported. Inter-scanner reproducibility of volunteer data in neurological [64] and abdominal [11] applications provides re-assurance that, with standardisation DW-MRI is suitable for use in multi-centre clinical trials.

## Data Storage and Analysis

### Data archiving, Transfer and Curation

A contemporary data archiving framework (termed a *Research PACS* [65]) needs to consider three important areas:

- A data storage platform that is resilient, secure and scalable and attached to multiple redundant servers. The *object store* is a currently popular example [66].
- A database and associated application program interfaces (APIs) for uploading, querying and downloading data. At present, so-called relational (SQL) databases dominate but the era of Big Data is seeing increasing use made of noSQL concepts.
- *User*-*facing components* that allow a user to access and interact with the data, e.g., a web browser interface and a toolkit of research applications.

The extensible Neuroimaging Archive Toolkit (XNAT), an open-source platform (Neuroinformatics Research Group, Washington University, St. Louis,MO,USA) has recently gained significant traction among academic groups as the foundation for such a Research PACS. However, several dedicated clinical trial management systems are also available commercially. Whichever product is used, standard operating procedures (SOPs) must be developed and used for staff training with both trial protocols and legislation vis-à-vis data handling.

**Fig. 3:**
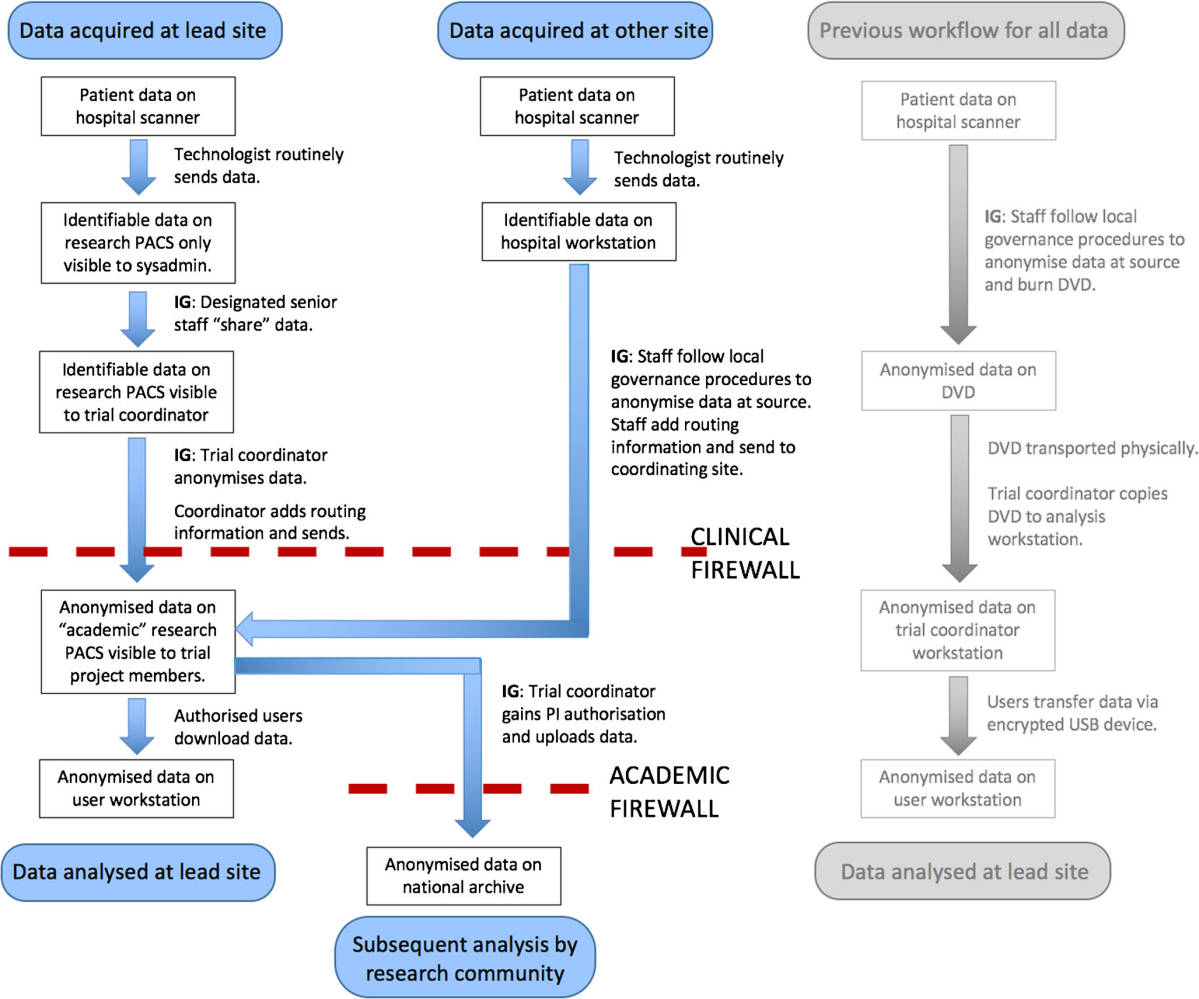
Data flow during a typical clinical trial curation process: Steps marked “IG” involve an information governance aspect, which will be determined by the ethics protocols attached to the trial. Local evaluation (not included as part of this trial workflow schematic) is a critical part of on-going patient care and is performed in context of clinical data, which centralized reading is not. The “research PACS” [65] referred to is provided by the eXtensible Neuroimaging Archive Toolkit (XNAT) [67]

Figure 3 presents a schematic of the workflow adopted within multicentre imaging trials. Clear organisation of multiple data types in a central hub brings significant time-savings when retrospective analysis is required [68] and all-electronic data transfer is now rapidly superseding the former practice of posting digital video discs (DVDs) containing trial images. Information governance is implemented via the use of designated staff who exercise a “gatekeeping” role. Data anonymisation by removal and/or replacement of metadata fields in the DICOM files requires a technical understanding of the processing to be done as well as knowledge of trial design and legal expertise. Data protection is achieved by designing robust systems, often including an element of geo-spreading, whilst prevention of unauthorised access is achieved by restriction on an IP address (implemented via appropriate firewall rules), user authentication and role definitions within database software. If a patient withdraws consent, it is possible to remove completely the data from the cohort used for ongoing analysis, but it is likely to prove impossible to remove these data from any summary statistics that have already been published, or any data record deposited as part of the publication process. Government bodies have guidelines pertaining to procedures required to ensure data integrity and compliance with information governance legislation [69].

### Software for image processing

As the variability of the measurement at low diffusion-weightings is high [70] and the signal decay is exponential, a low b-value of 100-150 s/mm^2^ is preferred when fitting a monoexponential function to derive ADC to reduce the influence of perfusion or flow effects on the measurement (Fig. 1B). Computed DW-MRI, (e.g., b=2000 s/mm^2^), improves DW-MRI contrast without any measurement penalty [71] but does not contribute to quantitation.

In DW-MRI, the use of non-mono-exponential models (stretched exponential, kurtosis, statistical and bi-exponential) [72–76] probe aspects of tissue microstructure [77] and differences between tumour sub-types or inter-tumour heterogeneity [78–83]. They may also provide an earlier indication of response to treatment than ADC estimates [84, 85]. Selection of the most appropriate model remains an area of active research: use of a model with many additional parameters risks over-fitting the data and may be sensitive to noise characteristics of the system rather than structural properties of the tumour or normal tissue. Vendor-supplied software to support calculation of these alternative diffusion attenuation models would help address some of these issues [77–86].

Finally, retention of tumour segmentations allows quality control (QC) review of data reduction procedures, as well as facilitating retrospective trial of alternative diffusion metrics drawn from the same 3-D segmentation objects stored at the pixel level [87]. As interobserver concordance is dependent on extent of sampling [88], the method of segmentation should be clearly recorded, for example, whether whole tumour or selected slices are segmented, and whether necrotic or cystic areas are excluded. A manual, semi-automated or automated method could also introduce variability in the measurement [89] and should be standardised.

## Maintaining quality standards across centres through the life of a trial

### QC and Data cleaning

Following set-up and Quality Assurance (QA), tests should be carried out at the beginning of the study to assess the baseline performance of each scanner, followed by regular QC tests over the course of the study (particularly after servicing and software upgrades) to detect changes in performance (Table 2). The frequency of tests and defined action limits, which specify the range of acceptable values may be study-dependent.

**Table 2:** Quality assurance and quality control considerations for imaging in multicentre clinical trials

	Quality Assurance (QA)	Quality Control (QC)
Why	To prevent errors and defects through planned and systematic actions	To identify and correct defects through a reactive process Benchmarking
When	Before trial activation	Over duration of trial
What	• Assure scanner calibration with a test object covering the desired range of ADC • Define minimal quality parameters needed to achieve required accuracy • Assure standardised acquisition by a master guideline • Assure correct acquisition before real patients by a human volunteer scan • Appropriate site training about all requirements and procedures and consider learning curves	• Control of data anonymisation and completeness • Control of data compliance to the imaging guideline - Limited control- randomly selected - Full control- all patients and all time points
How	• Implement standardized acquisition parameters that take account of variations in image geometry (anatomy, coverage) • Establish trial specific standard operating procedures (SOPs) • Establish trial management plan • Use a secure imaging platform accessible to named personnel at all trial sites	• Check scan quality with pre- defined criteria • Provide feedback to local sites - Retrospectively (by batch or at the end of the trial) - Prospectively (ongoing basis)

Within a multicentre trial, QA and QC procedures for imaging depend on the role of imaging in the trial [90]. Qualitative interpretation does not require the same level of QA/QC as for deriving quantitative data. The ROI size and number of pixels within it are crucial for quantitative assessments, particularly as many studies now address ADC distribution rather than mean or median values. Operational support for imaging QA and QC should be in place at trial setup and through the life of the trial (Table 2). A standardised and optimised acquisition protocol, which acknowledges vendor differences and incorporates acceptable and non-acceptable deviations should be defined and supplied to sites upfront. Acquisition of test data (test-objects, volunteers) reduces the likelihood of poor quality or non-evaluable imaging data being acquired from the first patient in the study; occasionally the first 1 or 2 patients may be considered as “run-in” to assess site compliance and data quality. From an ethical perspective, the intention must be for all included patients to contribute analysable data. However, if sites find it difficult to comply with the protocol, or if the first few patients' data are of poor quality, it may be necessary to discard those data following a protocol amendment to improve the methodology. Prospective QC with timely and informative feedback to the site enables supplementary correction to be taken and avoids non-assessable poor quality data at the end of the trial. Site upload of anonymised data via a web-based system requires training so that data are securely handled and correctly coded for inclusion in the trial imaging database.

### Assessing measurement variability

Measurement uncertainty arises from differences in acquisition (hardware and software differences between scanners as well as within scanners variations due to use of different protocols) plus post-processing parameters, longitudinal changes or ‘drift’ in MRI signal when using the same scanner over the study period as well as from natural physiological variation within and between study participants. The Radiological Society of North America (RSNA) Quantitative Imaging Biomarkers Alliance (QIBA) recommends that evaluation of biomarker reliability includes analysis of precision and bias estimation, plus measurement linearity, by comparison with an accepted reference or standard measurement [91]. For DW-MRI, *in vivo* physiological references are not available for bias/linearity measurements and these are extrapolated from phantom studies [20, 39, 91, 92].

Assessment of technical performance of an imaging biomarker includes measurement variability arising through differences between scanners (same patient, different scanners) [11], imaging protocols [93] and post-processing methods (such as different analysis software, lesion segmentation methodologies [94] and imaging readers [91, 92, 95]).

In trial design, the context in which the biomarker is being utilized dictates the measurement variations that must be accounted for. If measuring therapy-induced change, where it is usually possible to image each patient on the same scanner and for all analysis to be carried out by the same investigator, precision estimation is limited to repeatability [39]. For studies aimed at prognostication or lesion characterisation, ADC values will be compared between individuals or across institutions and as it is necessary to know whether a measured difference represents a true difference, measurement uncertainty including statistical appraisal due to reproducibility must be evaluated.

Coefficients of variation at different anatomic locations are in the range 3-10% [20, 96]. Inter-vendor two-site reproducibility coefficients of variation range from 14-27% [20]. In multicentre trials, a measured difference should be outside the 95% limits of agreement of the measurement uncertainty expected in a multicentre trial setting for it to be attributed to a true treatment-related difference. Alterations in lesion geometry also may affect segmentation thresholds and need consideration when making longitudinal measurements [97].

### Good Clinical Practice (GCP)

Clinical trials of investigational drugs and devices must comply with International Conference on Harmonisation GCP if they are intended to support regulatory approval [98]. For multicentre imaging studies, challenges exist in ensuring that different makes and models of MR scanner yield comparable data [90] and maintaining compliance with unfamiliar protocols at trial centres. The Food and Drug Administration has specific guidelines to help ensure that imaging biomarkers are measured in accordance with the trial’s protocol [99], and that quality is maintained over time and between sites: it recommends that sponsors employ an “Imaging Charter”, ancillary to the trial protocol, which defines the imaging process in exhaustive detail. Sponsors often engage specialist Imaging Clinical Research Organisations to perform site qualification and training, phantom-based QA/QC, pilot studies, data management and analysis. Double baseline studies are valuable in verifying repeatability [100], although the additional burden may deter patients, sponsors and ethical committees.

### Reporting considerations for clinical governance

Performing imaging in clinical trials risks discovery of incidental findings (IFs) that may require action and, therefore, require review by a trained diagnostician [101]. Ethical and legal issues surrounding IFs are a key element of the duty of care owed by researchers to study participants (Table 3). Generic recommendations are offered by the National Institute of Health in the USA and Royal Colleges in the UK (Table 3). No specific recommendations have yet been proposed for studies utilising DW-MRI.

**Table 3:** Recommendations for dealing with Incidental Findings

Questions arising from research scan	NIH recommendation (reproduced from Wolf [88])
Do researchers have an obligation to examine their data for IFs?	‘It is unrealistic to place on researchers an affirmative duty to search for IFs’
What should be done if an IF is detected - should it prompt specialist referral for definitive diagnosis?	‘Obligation to establish a pathway for handling IFs and communicate that to the Independent ethics committee/review board and research participants’
What should the research participant be told?	‘In many, but not all circumstances, researchers have an obligation to offer to report IFs to participants’
What should research protocols and consent forms include relating to IFs, should the right to refuse knowledge of IF be addressed?	‘Researchers have an obligation to address the possibility of discovering IFs in their protocol and communications with the IRB, also in consent forms and communications with research participants’
Key NIH recommendations for addressing IFs: • Plan for the discovery of IFs in study protocol and IRB communication • Plan to verify and evaluate a suspected IF with expert review if necessary • Researchers and IRBs should create and monitor pathways for IFs • Address IFs in the consent process • Plan to determine whether to report IFs, based on likely health importance: a. Strong net benefit to health from reporting IF b. Possible net benefit c. Unlikely net benefit • Address the potential for IFs in future analyses of archived data	

A report of whole body DW-MRI in healthy volunteers has shown IFs in 29% of subjects. Of these 30.6% were considered of ‘moderate significance’ and 10.2% ‘high significance’, requiring specialist review but only a minority of scans required further action [102]. In myeloma, IFs were seen in 38% (67/175) of examinations, 20% of findings were equivocal and after specialist radiologist and clinical review, only 3% of cases prompted further investigation. It is mandatory to introduce an image review process, triage and referral pathways embedded into trial design and reflected in consenting procedures. For multicentre trials, this system should account for the logistical hurdles that arise due to data storage and delays in data viewing. For cases where data are interpreted centrally, procedures should define a reporting mechanism, so that IFs discovered centrally prompt action locally.

## Proposals for future workflow

A summary of factors that need to be addressed to ensure that ADC is accurate and reproducible across multiple centres together with recommended actions is given in Table 4. Consideration of these enable guidelines and drug approvals to be written and implemented consistently so that repeatability is smaller than the clinically-significant changes sought in a clinical trial or trial-of-therapy [103]. MR instruments must be designed and maintained so that selected diffusion-weightings are imposed faithfully, sufficient gradient strengths must be provided to allow adequate diffusion-weighting where *T*_2_ is short, pulse sequences, k-space trajectories and analysis modules must be integrated, the number of measurements (signal averages) optimised, nomenclature standardised and technical details retained in public DICOM image fields.

**Table 4:** Summary of factors contributing to ADC variability in multicentre trials and measures required to reduce them

Factors affecting multicentre DW-MRI variability	Steps to reduce ADC variability
Low SNR of data	Higher field strength, receiver technology (arrays), digital compensation schemes, optimal sequence parameters (including b-values), increased signal averages, interpolation of single pixels/voxels
Image distortion	Eddy current compensation, improved B0 homogeneity (shimming), increased bandwidth, lower b-values, reduced ETL and matrix
Ghosting artefacts	Adjust receiver bandwidth and echo-time
Motion artefacts	Breath-hold, respiratory triggering, cardiac triggering, antiperistaltic agents if necessary
Statistical errors due to region of interest size	Specify a minimum lesion size for inclusion into the trial; specify ROI size, increase signal averages
Quality Assurance measures	Standardised test objects, standardised operating procedures for their use and pass/fail criteria
Test-retest repeatability data	Build test-re-test baseline scans into trial protocol for a subset of patients at each site
Quality Control measures	Longitudinal review of repeated test object data from each site for the duration of the trial
Data Transfer, Curation and access	Dedicated server and written standardised procedures within the trial protocol for data anonymisation, transfer to dedicated software platform and access by trial researchers
Image processing methodology	Robust standardised software (preferably FDA approved or CE marked) that can be accessed by observers from multiple sites to validate reproducibility of results. Standardised segmentation methods (2-D or 3-D, inclusion/exclusion of necrotic areas, manual vs semi- automated or automated ROI definition)

Once the reliability of the ADC has been established, tumour heterogeneity of the biomarker may provide further opportunity for tumour mapping (spatial display of quantitative parameters) to guide surgery or radiotherapy. Locations above (or below) a cut-off may be selected for targeting. There is some regulatory precedent for such a workflow with the US approval of [^99m^Tc]-tilmanocept uptake above cut-off as a biomarker for surgical removal of lymph nodes in patients with breast cancer or melanoma [104]. Again, as a prognostic or predictive biomarker, it may be the proportion of the tumour above (or below) an ADC cut-off which is of interest, just as with hypoxia biomarkers [105], rather than the average across a tumour. For acute response biomarkers and trial-of-therapy biomarkers, a more ambitious workflow is functional diffusion mapping [97, 106], which attempts to correlate changes voxel-wise between baseline and follow-up. This approach requires that specific voxels at baseline correspond to specific voxels at follow-up, an assumption which may be difficult to validate.

It is unlikely that ADC will find a decision-making role in healthcare until vendors incorporate adequate ADC reliability into scanner maintenance (just as RECIST relies on dimensional accuracy verified by scanner maintenance). However, vendors are unlikely to consider that it is a good use of their resources to provide and maintain accurate ADC measurements until there is a demand from their customers, the radiologists; these radiologists are unlikely to demand accurate ADC measurements until there is an evidence base from multicentre trials to show the impact of ADC measurements on health outcomes, and such an evidence base is difficult to collect unless scanners routinely generate accurate ADC measurements. Expert groups and consortia such as QuIC-ConCePT, EIBALL (European Biomarkers Alliance), NCI-QIN (Quantitative Imaging Network) and QIBA are essential in supporting standardisation to break us out of this vicious circle and enable ADC quantitation to enter clinical workflows.

In conclusion, the use of ADC as an imaging biomarker in multicentre trials demands processes that standardise data acquisition and analysis within a framework of Quality Assurance and Quality Control. Test-object and healthy volunteer studies should be used to develop an imaging protocol for multi-vendor, multi field-strength use and establish the accuracy of the ADC measurement. Finally, data storage in a central trial repository ensures traceability as well as data preservation for further research.
